# Implementation of a Robotic Hand Glove in the Physiotherapy Treatment of Carpal Tunnel Syndrome Secondary to Volar Barton Fracture: A Case Report

**DOI:** 10.7759/cureus.55402

**Published:** 2024-03-02

**Authors:** Vaishnavi M Thakre, Pratik Phansopkar

**Affiliations:** 1 Musculoskeletal Physiotherapy, Ravi Nair Physiotherapy College, Datta Meghe Institute of Higher Education & Research, Wardha, IND

**Keywords:** case report, carpal tunnel syndrome, robotic hand glove, physiotherapy, volar barton fracture

## Abstract

A volar Barton fracture is a compression injury that usually results from a fall onto an outstretched, pronated wrist. It is characterized by an intraarticular marginal volar shearing fracture of the distal radius. Despite the prevalence of distal radius fractures, consensus regarding optimal treatment remains challenging. To achieve adequate alignment, a variety of treatment techniques are available, including open reduction with plate and screw fixation, external fixation, and closed reduction with pinning. Regardless of the method of treatment, carpal tunnel syndrome (CTS) is the most prevalent complication that is commonly seen in distal radius fractures. Establishing an effective post-operative rehabilitation regimen, primarily comprising functional physiotherapy interventions, is vital to managing this condition. This case report discusses the management of acute CTS secondary to a volar Barton fracture, characterized by symptoms including pain, diminished strength and mobility of the wrist joint, and impaired grip strength and fine motor skills. The outcome measures utilized were the Upper Extremity Functional Index and the Boston Carpal Tunnel Syndrome Questionnaire. A customized physical therapy regimen was implemented, comprising cryotherapy, range of motion exercises, and grip strengthening utilizing a robotic glove. This tailored approach proved effective in promoting early functional recovery and improving activities of daily living.

## Introduction

There are a variety of distal end fractures of the radius, depending on the bone quality, age, and mechanism of injury. These fractures include variations such as the dorsal and volar Barton, Smith, and Colles fractures [1]. Since they account for 75% of forearm fractures and 17% of all fractures, fractures around the lower end of the radius are the most common upper extremity fractures encountered in clinical practice. The range of incidence rates per 10,000 people per year is 5.7-124.6 people [2]. John Rhea Barton, an orthopedic surgeon in Philadelphia, was the first to describe the Barton fracture. It is characterized by a distal radius fracture that passes through the dorsal portion of the articular surface; the radiocarpal joint is frequently dislocated as a result [3]. The Barton fracture is distinctive among distal radius fractures and dislocations due to the fact that it maintains intact contact between the radius and carpus as the radiocarpal ligament is not disrupted [4]. According to the Arbeitsgemeinschaft Osteosynthese (AO) classification system, distal radius fractures resulting from shearing force are categorized as type B fractures. Specifically, B3 fractures denote volar Barton fractures. These B3 fractures are then subcategorized based on the size of the volar fragment into B3.1 for small fragment fractures, B3.2 for large fragment fractures, and B3.3 for comminuted fractures [5].

Comminuted distal radius fractures are often caused by a fall onto the outstretched hand from a standing height. However, it is crucial to emphasize that a minority of patients may experience substantial energy-related damage as a result of this mode of injury [6]. Individuals with Barton fractures frequently seek medical attention in urgent care when presenting with deformity, edema, and acute wrist pain as a result of recent trauma. Tenderness, ecchymosis, and joint swelling are common findings during a physical examination of the wrist. Pain typically results in a limited range of motion (ROM) for the wrist joint [7]. The initial assessment of a Barton fracture commences with wrist radiographs, typically comprising lateral and frontal views. Additionally, oblique views of the wrist may be acquired to aid in diagnosis. Anteroposterior radiographs are utilized to determine radial height inclination, articular step, and ulnar variance. Meanwhile, lateral radiographs allow detection of comminution, coronal split, and volar tilt [8]. Nonoperative management typically yields unsatisfactory outcomes and is accompanied by potential complications, including deformity, early-onset osteoarthritis, instability, and subluxation [9]. Several surgical approaches have been reported in the medical literature for the management of volar Barton fractures. These include percutaneous Kirschner pinning and closed reduction with external fixation, as well as open reduction with volar buttress plating, which is currently recommended for the management of such fractures. These procedures often produce favorable realignment and immediate stability. Additionally, they enable expedited wrist mobilization, potentially mitigating the risk of wrist stiffness [10].

Post-traumatic osteoarthritis, weakened grip strength and endurance, carpal instability, and restricted motion are all sequelae of articular malalignment after distal radius fractures. Carpal tunnel syndrome (CTS) is the most frequent nerve-related complication, and it occurs often in distal radius fractures regardless of treatment mode [11]. Internal fixation for distal radius fractures is often justified by the possibility of improved wrist motion and function from early mobilization and grip strengthening, similar to outcomes observed with other periarticular fractures. [12]. Another innovative way to support rehabilitation therapy is the development of robotic gloves for hand rehabilitation therapy. A robotic glove prosthesis specifically designed for hand rehabilitation in patients with grip disorders is presented in this scholarly publication [13]. In order to restore hand strength and movement, repetitive hand movements are frequently employed as a rehabilitation strategy. A wearable hand rehabilitation gadget can improve the quality of rehabilitation activities and allow physiotherapists to extend their training [14]. Our aim was to evaluate the effect of utilizing a robotic glove in order to achieve early wrist movement and grip strength to gain early fine movement in the case of acute CTS secondary to a volar Barton fracture.

## Case presentation

Patient information

**Figure 1:**
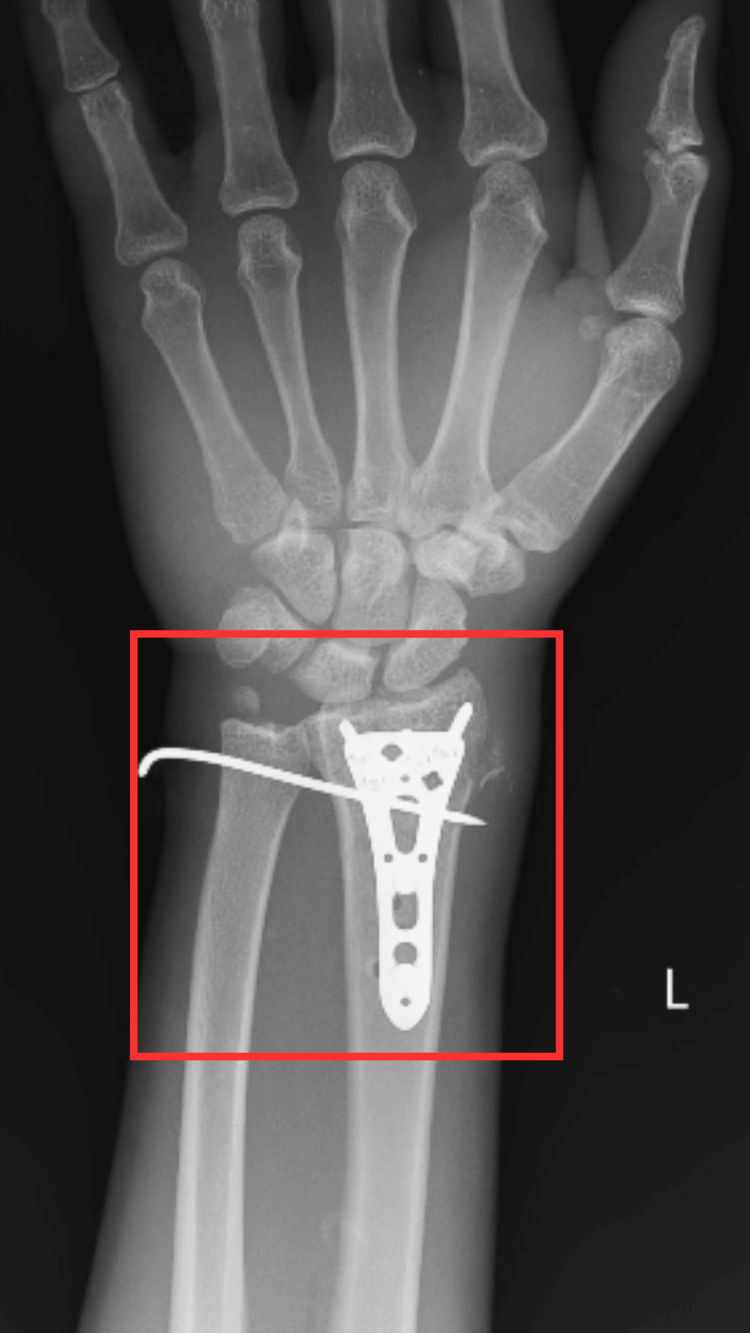
X-ray of the wrist joint The square shows ORIF with plate osteosynthesis for a volar Barton fracture. ORIF, open reduction and internal fixation

We report the case of a 45-year-old male who was apparently alright two months ago when he had a road traffic accident and fell from a two-wheeler, after which he experienced pain and swelling of the left wrist. Pain was sudden in onset, gradually progressive, sharp shooting in nature, constant throughout, increased in intensity on movement, and decreased on rest and immobilization. Immediately after the trauma, the patient came to our tertiary care hospital, where dressing was done, and he had undergone an X-ray investigation that revealed a B3.3 (according to the AO classification) volar Barton fracture on the left side. He underwent open reduction and internal fixation (ORIF) with plate osteosynthesis for a volar Barton fracture (Figure 1). After surgical repair, the patient developed acute CTS and pain at the wrist joint, reduced strength and range of the wrist joint, and a loss of grip strength, for which a tailor-made physiotherapy regimen was started.

Physiotherapy assessment

Prior to initiating the examination, the patient provided informed consent. He was cooperative, conscious, and oriented to place, person, and time. Upon examination, he was hemodynamically stable. The patient had a mesomorphic build with a BMI of 29 kg/m^2^. Visible swelling was noted near the wrist joint. Palpation revealed Grade 2 tenderness based on the tenderness grading system. The patient reported dull pain, rated 8/10 during activity and 3/10 at rest on the Numerical Pain Rating Scale. The ROM and strength were notably diminished at the wrist joint of the left upper limb.

Physiotherapy intervention

**Figure 2:**
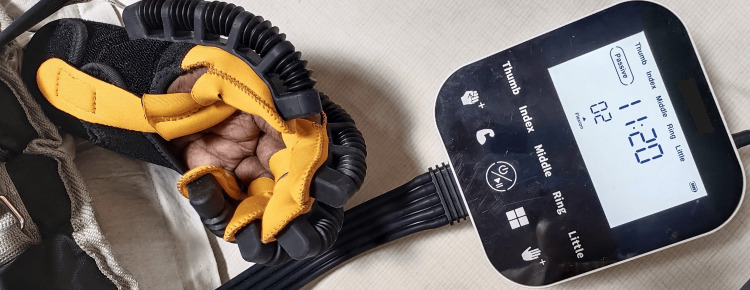
Patient performing passive ROM exercises for the wrist joint using a robotic glove ROM, range of motion

Based on the patient’s clinical state, the physiotherapist designed tailored exercise sessions. The treatment duration was four weeks. Table 1 and Figure 2 depict the physiotherapy protocol.

**Table 1:** Physiotherapy intervention AAROM, active assisted range of motion; AROM, active range of motion; PROM, passive range of motion; ROM, range of motion; reps, repetition

Week	Goals	Intervention	Repetitions	Rationale
Weeks 1-2 (during plaster cast)	To prevent edema	Hand elevation above heart level	Twenty reps × one set (two times per day)	Mobility will be restored by lowering edema and swelling
	To maintain the strength of the intrinsic muscles of the hand and wrist musculature	Isometrics: intrinsic muscles of the hand (week 1) and wrist flexors and extensors (week 2)	Ten reps × one set (two times per day)	Prevents muscle wasting
	To maintain the strength and ROM of the unaffected limb	AROM exercises for unaffected limb	Ten reps × one set (10-second hold)	Prevents joint stiffness
Weeks 3-12 (after removal of the plaster cast)	To reduce pain	Cryotherapy	Duration: seven minutes twice daily	Efficient pain control facilitates patient mobility and engagement in physical activities, thereby promoting the healing process
	To improve strength for wrist and hand muscles	Gentle resistive exercises for the digits and wrist (weeks 3-7)	Ten reps × one set (two times per day)	Contractions of the muscles against resistance cause an “overload” phenomenon, which stimulates the muscular system to adapt and gain training effects
		Progressive resistive exercises to the wrist, digits, and all groups of muscles (weeks 8-12)		
	To improve ROM, wrist joint, and grip strength	Isometric exercises for wrist flexors, extensors, and radial and ulnar deviators	Ten reps × one set (10-second hold)	By gradually increasing their flexibility and ROM, patients can improve their physical function and progress toward the restoration of their autonomy
		Supination and pronation are encouraged. Active ulnar deviation and radial deviation using a robotic glove		
		Pain-free PROM, AAROM, and AROM exercises for the wrist joint using a robotic glove	Ten reps × one set (two times per day)	
		Neural mobilization		

Follow-up and outcome measures

For four weeks, the patient underwent a structured physical therapy regimen, followed by a subsequent follow-up evaluation. The findings of the outcome measure are shown in Tables 2-4.

**Table 2:** Wrist MMT 2, full ROM with gravity eliminated; 4, full ROM against gravity, moderate resistance MMT, manual muscle testing; ROM, range of motion

Muscle group	Pre-treatment MMT	Post-treatment MMT
Wrist flexors	2	4
Wrist extensors	2	4
Pronators	2	4
Supinators	2	4
Ulnar deviator	2	4
Radial deviators	2	4

**Table 3:** Wrist ROM ROM, range of motion

Movement	Pre-treatment active ROM	Post-treatment active ROM
Wrist flexion	30^o^	80^o^
Wrist extension	20^o^	75^o^
Pronation	10^o^	75^o^
Supination	15^o^	80^o^
Radial deviation	5^o^	25^o^
Ulnar deviation	8^o^	30^o^

**Table 4:** Outcome measures NPRS, Numerical Pain Rating Scale; UEFI, Upper Extremity Functional Index

Outcome measures	Pre-intervention	Post-intervention
UEFI score	42	72
Boston Carpal Tunnel Syndrome Questionnaire	Symptom severity: 3	Symptom severity: 0
	Functional status: 4	Functional status: 1
NPRS	On activity: 6/10	On activity: 3/10
	On rest: 2/10	On rest: 1/10

## Discussion

Even though volar Barton fractures are not uncommon, the optimal treatment approaches for them are still up for debate. The closed reduction with percutaneous Kirschner wire fixation and external fixation or open reduction with buttress plating has garnered significant support at this time [15]. In this study, we have discussed a case of a 45-year-old male diagnosed with acute CTS secondary to a volar Barton fracture and managed surgically with ORIF with plate osteosynthesis. The ORIF led to post-operative pain and loss of wrist proprioception, ROM, and strength, for which he had undergone physiotherapy management, including robotic gloves to regain early grip strength, which proved to be effective in early functional recovery.

We incorporated robotic gloves into our treatment, which helped in improving strength and ROM of the wrist and fingers, which led to early grip strengthening and regaining fine movements, which also led to an improved score on the upper extremity functional index and Boston Carpal Tunnel Syndrome Questionnaire. Biggar and Yao also stated that robotic exoskeletons are an emerging way of supporting physical therapy, owing to their capacity to provide a stable structure that improves patient posture. Furthermore, they provide a strong foundation for positioning the components that direct and support movements [16]. A wearable soft robotic device holds promise for enhancing the benefits of rehabilitative therapy by offering enhanced portability, improved affordability, simplified tailoring, reduced weight, grip-strengthening capabilities, safer interactions between humans and robots, expanded ROM, and the ability to conduct task-specific training or exercises simulating activities of daily living (ADLs) [17].

According to Kochar and Samal, for those with volar Barton fractures, physiotherapeutic therapies such as ROM exercises, cryotherapy, and muscle-strengthening exercises can be administered [18]. Additionally, exposure to cold decreases nerve transmission velocity and vasoconstriction, which, in turn, reduces blood flow and relieves pain and edema [19]. In this case, we have seen that the patient has undergone physiotherapy treatment that included isometric and active ROM exercises using a robotic glove, cryotherapy, strengthening exercises, and neural mobilization, which increased wrist grip strength and ROM, reduced pain, and regained fine movements, which led to improved ADLs and early functional recovery.

## Conclusions

Dealing with a volar Barton fracture unveils significant challenges and can result in various secondary consequences, such as CTS. Physiotherapy plays a pivotal role in the rehabilitation of individuals with CTS secondary to volar Barton fractures. Wearable hand rehabilitation devices can aid physiotherapists in promoting early wrist mobility and grip strength, facilitating early fine motor movement, and improving the efficacy of rehabilitation exercises. Physiotherapeutic interventions, including cryotherapy, neural mobilization, ROM exercises using robotic gloves, and muscle strengthening exercises, are beneficial for individuals experiencing acute CTS secondary to volar barton fractures.
